# Clinical profiles, referral patterns and low vision aid prescribing across four rehabilitation centres in Ghana: protocol for a multicentre retrospective record review

**DOI:** 10.3389/fmed.2026.1936778

**Published:** 2026-10-09

**Authors:** Kwadwo Owusu Akuffo, Josephine Ampong, Josephine Ampomah Boateng, Caleb Ofosu Yeboah, Lovinger Ato Quansah, Richard Atakora Baafi, Edward Aboagye Yeboah, Isaiah Osei Duah Junior, Sylvia Agyekum, Sylvester Kyeremeh, David Ben Kumah, Albert Kwadjo Amoah Andoh, Emmanuel Tettey Doku, Gabriel Kwaku Agbeshie, Samuel Oteng Brako, Kenneth Bempah Karikari, Nana Akwasi Owusu Mensah, Eldrick Adu Acquah, Ellis Sarpong Mainuh, Maame Abena Kesewah Boaitey, Bright Owusu Prempeh, Louis Oteng-Gyimah, Afua Asantewaa Nkansah, Patience Boatemaa-Twum, Henry Owusu Bota, Josephine Boakye Owusu, Golda Dokuaa Kwapong, Nicholas Sarpong, Hornametor Yao Afake, Werner Eisenbarth

**Affiliations:** 1 Department of Optometry and Visual Science, College of Science, Kwame Nkrumah University of Science and Technology, Kumasi, Ghana; 2 University Hospital, Eye Clinic, Kwame Nkrumah University of Science and Technology, Kumasi, Ghana; 3 Anglican Eye Clinic, Jachie, Ghana; 4 Tafo Government Eye Clinic, Tafo, Ghana; 5 Presbyterian Hospital Eye Clinic, Agogo, Ghana; 6 Department of Psychology, John R. and Kathy R. Hairston College of Health and Human Sciences, North Carolina Agricultural and Technical State University, Greensboro, NC, United States; 7 Asante Akyem Central Municipal Hospital, Konongo, Ghana; 8 Seventh day Adventist Hospital, Bremang, Ghana; 9 Suntreso Government Hospital, Suntreso, Ghana; 10 Kuntenase Government Hospital, Kuntenase, Ghana; 11 Global Evangelical Mission Hospital, Awonmaso, Ghana; 12 National Eye Care Unit, Ghana Health Service, Accra, Ghana; 13 Department of Applied Science and Mechatronics, HM Hochschule München University of Applied Sciences, Munich, Germany

**Keywords:** low vision, patient outcomes, rehabilitation, residual vision, service delivery, vision aids, visual impairment

## Abstract

**Background:**

Low vision, defined as best-corrected visual acuity of less than 6/18 in the better eye or a visual field constriction of 20° or less, is a condition in which residual vision may be optimized through optical and non-optical aids. Despite the importance of low vision services in maximizing residual vision and supporting rehabilitation and quality of life, evidence on patient profiles and service delivery in Ghana remains limited. The HM KNUST Low Vision Project aims to address this service gap through a multicentre retrospective medical record review across four centres, characterizing patient profiles, referral patterns, and low vision aid prescribing to inform the strengthening of low vision services in Ghana.

**Methods:**

The HM KNUST Low Vision Project is a collaboration between the Department of Optometry and Visual Science at Kwame Nkrumah University of Science and Technology, Ghana, and the Department of Applied Sciences and Mechatronics at Munich University of Applied Sciences, Germany. As part of this collaboration a comprehensive low vision patient form was developed in collaboration with Ghanaian optometric stakeholders and distributed to clinicians across selected pilot eye care facilities. The form captures biodata, systemic health, visual characteristics, referral patterns, and low vision aid prescriptions to facilitate standardized data collection across centres. Descriptive statistics will summarize patient characteristics, clinical profiles, referral patterns, and low vision aid prescribing. Inferential analyses will assess associations between patient and clinical characteristics, referral patterns, and prescribing patterns across participating centres.

**Discussion:**

This study will provide multicentre evidence on low vision service delivery in Ghana, offering insights to guide rehabilitation services, clinical decision-making, and service planning. The standardized patient form will enable consistent characterization of patient needs, referral pathways, and low vision aid prescribing across centres, establishing an important foundation for improving the accessibility, consistency, and responsiveness of low vision care in Ghana.

## 1 Introduction

Imagine an older adult who can recognize people across a room but struggles to read medication labels, navigate unfamiliar places, or perform routine household tasks because of reduced vision. Similar challenges occur across the life course, with children struggling to read classroom materials and working-age adults unable to perform routine tasks at work such as driving to work or reading office documents from a desktop computer. These everyday challenges reflect the broader burden of low vision, which affects over 295 million people worldwide (1, 2), including an estimated 14.31–29.16 million people living in regions with low to middle Socio-Demographic Index scores, such as sub-Saharan Africa (SSA) (3), where a pooled meta-analytic estimate from Ghana indicates that 5.79% of the population, approximately 1 in 17 people, live with moderate to severe visual impairment (4).

As the illustrative example shows, low vision extends beyond visual acuity to affect activities of daily living and quality of life. For example, children with residual vision suffer from delayed social development (5, 6) that culminates in lower self-esteem (7, 8) and challenging educational discourse (6, 9). Similarly, among working-age adults, diminished visual function predisposes them to difficulties securing employment, impaired mobility, and financial difficulties, which hinder their ability to perform family responsibilities (10–12). Older adults may experience greater challenges with activities of daily living, mobility, and social participation, with increased dependency (13, 14). In these circumstances, individuals may adopt multiple strategies to cope with visually challenging situations, such as modifying daily routines (15), relying on family or social support (16), using assistive devices (17, 18), or adopting alternative strategies to perform everyday tasks (15). Despite these adaptations, the challenges associated with low vision can negatively affect emotional well-being, life satisfaction, and overall quality of life (QoL) (19–21).

One useful strategy to address these challenges is to provide robust low vision services (22), which constitute low vision rehabilitation, a multidisciplinary medical process characterized by the assessment and optimization of residual vision to improve functional ability and independence among individuals with irreversible visual impairment (23, 24). Low vision rehabilitation is therefore regarded as a valuable intervention for enhancing daily functioning and QoL (25, 26). Yet, efforts to address visual impairment in Ghana have focused predominantly on curative services for avoidable blindness, particularly age-related cataract and uncorrected refractive errors (27, 28), with limited attention to those with residual vision (29). Despite the positive impact of low vision services on treatment outcomes, access to these services is limited and impaired by a range of challenges at both the patient and health system level (29, 30). For instance, at the patient level, there is a lack of awareness of available services, denial of need for low vision aid, lack of transportation, lack of referrals, misconceptions of the service, negative societal views, and the high cost of low vision devices (22, 29–31). Similarly, at the health system level, barriers include limited availability of trained eye care providers and optical aids, as well as inadequate referral criteria and clinical pathways for directing patients to specialized low vision care (29, 30, 32). Consequently, patients may not only be unable to access appropriate low vision services but also miss opportunities for vision rehabilitation, which may improve visual function and QoL (33).

The World Health Organization has outlined a structured model for low vision services across primary, secondary, and tertiary levels of care for both children and adults, but no previous research in Ghana has systematically examined this model in practice. It is within this context that the HM KNUST Low Vision Project was established as a collaborative initiative between the Hochschule München (HM) University of Applied Sciences in Germany and the Kwame Nkrumah University of Science and Technology (KNUST) in Ghana (34). This project applies a hierarchical systems approach to understand and strengthen the low vision care ecosystem by integrating stakeholders, patients, and eye care providers into a coordinated model of service delivery. Central to the project is the establishment of a tertiary low vision centre at KNUST University Hospital, complemented by the phased development of three secondary and six primary low vision centres across the Ashanti region to create a fully functional referral network. In addition to service expansion, the project emphasizes professional capacity-building through continuing education programs, the development of standardized low vision referral documents, and the creation of an online learning platform to support sustained workforce training.

### 1.1 Study rationale and novelty

To the best of our knowledge, no previous study in Ghana has assessed low vision service delivery across multiple rehabilitation centres at different levels of care. The present study uses routinely collected clinical data from four HM KNUST low vision centres operating at secondary and tertiary levels of care. Unlike previous single-centre assessments (33), the multicentre design will allow characterization and comparison of the demographic and clinical case mix across participating centres and examination of differences in referral patterns and service provision. The study will also assess the geographic distribution of patients and referring facilities, providing insight into referral patterns and the areas served by individual centres. In addition, routinely collected prescribing and dispensing data will enable examination of patterns in low vision aid recommendation and dispensing, including the types of optical and non-optical aids provided and variations across centres. Because the dataset includes dates of service, the study will also describe patterns in service volume over time, including changes in patient attendance and service utilization across participating centres. Together, these analyses will provide a broader systems-level description of low vision service delivery in Ghana than has previously been available.

Therefore, this protocol aims to provide a systematic framework for documenting and evaluating low vision service delivery and associated patient outcomes across four HM KNUST low vision centres in Ghana. Specifically, it will describe the demographic and clinical profile of low vision cases presented to these centres, characterize the referral pathways, examine patterns of low vision aid prescribing and dispensing, and evaluate changes in service volume over time. Through standardized data collection and analysis, this protocol will generate evidence to inform quality improvement efforts, guide resource allocation, identify low vision training needs, and contribute to the broader knowledge base on low vision care in African settings.

### 1.2 Aim

To describe the demographic and clinical characteristics of patients accessing low vision services, evaluate patterns of referral and low vision aid provision, and examine factors associated with prespecified clinical and service outcomes across four centres in Ghana.

### 1.3 Objectives

1. To describe the demographic and clinical profile of patients presenting with low vision to the HM KNUST secondary and tertiary low vision centres.
2. To characterize and classify the underlying causes of low vision among patients attending the participating centres.
3. To describe patterns of referral to the network, and compare the demographic and clinical case mix of patients across participating centres.
4. To describe the types of low vision aids recommended/prescribed and dispensed, and examine factors associated with aid prescription and visual performance.
5. To examine patterns of service utilization across the participating centres, including differences in patient attendance and service volume over time.

## 2 Methods

### 2.1 Study design

This study will employ a multicentre retrospective record review design, using routinely collected clinical records from three secondary and one tertiary HM KNUST low vision centres from April 1, 2023 to April 30, 2026. The unit of analysis for all primary and secondary endpoints will be the individual patient, represented by the index low vision assessment during the study period. The index assessment will be defined as the first eligible low vision assessment recorded for a patient during the study period. In this study, low vision is defined as best-corrected visual acuity in the better-seeing eye worse than 6/18 but equal to or better than 3/60, consistent with WHO (ICD-10) classification (35).

### 2.2 Study setting and centre responsibilities

The study is embedded within the HM KNUST Low Vision Network, a structured, tiered service system operating across the Ashanti Region of Ghana. The network comprises ten facilities including one tertiary, three secondary, and six primary facilities (36). The primary facilities serve as the initial points of contact, providing low vision screening and identifying patients who may require further assessment. Patients requiring specialized care are referred to the secondary and tertiary facilities, where comprehensive low vision assessments are conducted. These secondary and tertiary facilities will also serve as the study's data collection sites, enabling standardized assessment and documentation of patients referred for comprehensive low vision care (see Table 1 and Figure 1).

**Table 1:** Clinical responsibilities of the three service tiers within the HM KNUST Low vision network.

Centre level	Opening date	Service availability	Staffing	Tests available	Devices available	Follow-up provision	Data included in study?
Primary (*n* = 6)	March 2026	-Identification of patients with suspected low vision -Referral of low vision patients to either the tertiary or secondary centers.	Ophthalmic nurses	Distance and near visual acuity measurement (LogMAR)	Non-optical aids	Yes — (after secondary or tertiary intervention)	No — screening data was not extracted for this study
Secondary (*n* = 3)	January 2025	-Full low vision assessment -Prescription and dispensing of optical and non-optical low vision aids -Management of moderate-complexity cases -Referral of complex cases to the tertiary centre	Optometrist (trained in low vision assessment)	Distance and near BCVA, contrast sensitivity, color vision, depth perception, visual field, refraction	Optical and non-optical low vision aids	Yes — return visits managed at same centre	Yes — patient-level records extracted
Tertiary (*n* = 1)	December 2024	-All functions of secondary centers, plus: -Management of complex and specialist cases -Multidisciplinary rehabilitation -Advanced optical and electronic aid assessment and dispensing -Low vision training center	Optometrists, multidisciplinary team	Distance and near BCVA, contrast sensitivity, color vision, depth perception, visual field, refraction, advanced visual function tests	Optical, non-optical, and electronic low vision aids	Yes — return visits managed at same centre	Yes — patient-level records extracted

**Figure 1:**
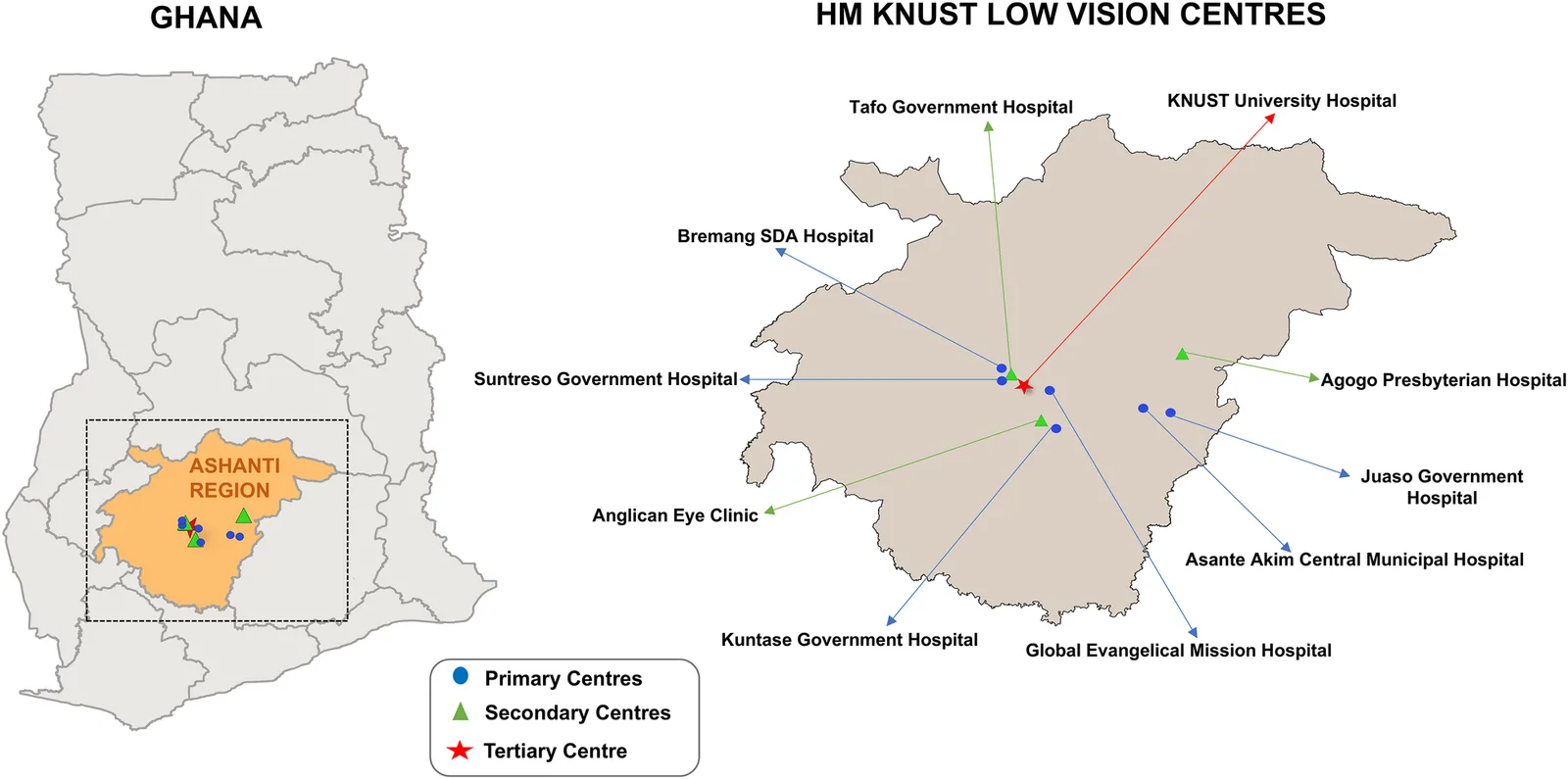
Distribution of the HM KNUST Low vision centers across Ashanti region, Ghana. Data source: facility coordinates recorded via Google Maps (2026); Map source: GADM v4.1 boundaries. Produced in QGIS 3.44.5; Only secondary and tertiary centres contribute patient-level data.

### 2.3 Participants and eligibility criteria

The study population comprises all patients seen at the four data-collecting centres who underwent a low vision assessment during the study period.

#### 2.3.1 Inclusion criteria

1. All patient records from the three secondary and one tertiary HM KNUST low vision centres from April 1, 2023 to April 30, 2026.
2. Confirmed diagnosis of low vision (BCVA < 6/18 and ≥ 3/60 in the better eye) for both children and adults.
3. Patients with documented cerebral or cortical visual impairment who underwent low-vision assessment or rehabilitation will be included and analysed as a separate clinical subgroup, irrespective of whether they meet the predefined visual-acuity threshold for low vision.
4. Patients with documented functional rehabilitation needs, including those with visual field loss, will remain eligible despite visual acuity better than the distance threshold.
5. Records containing minimum best-corrected visual acuity (BCVA) in the better-seeing eye, together with age, sex, and primary diagnosis.

#### 2.3.2 Exclusion criteria

1. Duplicate records representing the same patient and index assessment.
2. Records with fewer than three completed data fields, where the available information is insufficient to establish eligibility or provide meaningful clinical information.
3. Records of patients with no light perception (NLP) will be excluded from the primary analytic population only when there is no documented rehabilitation need or intervention.

### 2.4 Data collection

#### 2.4.1 Data collection instruments

Data collection at all four sites will be carried out using the standardized HM KNUST low vision patient form, which was developed collaboratively by the HM KNUST project team for use across the network and was distributed to and routinely used at the secondary and tertiary centres before commencement of the study. It was developed with reference to existing low-vision assessment tools (37, 38) and informed by relevant literature and clinical guidelines (39–41). See Supplementary Material S1.

#### 2.4.2 Data collection procedures

Data will be obtained from the existing HM KNUST patient forms completed during low vision assessments at the four participating centres. The patient form captures patient demographic and clinical information, needs assessment, visual function measurements, diagnosis, low vision aid recommendations, and dispensing information. The optometrist at each participating centre enters the information from each completed patient form into a restricted-access Google Sheets database shared only with authorized members of the research team.

The resulting dataset will be reviewed for completeness, consistency, duplicate entries, and data-entry errors before analysis. Missing, unclear, or undocumented information will be coded accordingly and will not be inferred. Where clarification is possible from the original patient form, the information will be verified before coding.

### 2.5 Clinical measurement procedures

#### 2.5.1 Distance visual acuity

Distance visual acuity was assessed using the Early Treatment Diabetic Retinopathy Study (ETDRS) visual acuity chart in an ETDRS Illuminator Cabinet at 4 m. Measurements were recorded separately for the right eye, left eye, and both eyes where available. The clinical record will indicate whether measurements were presenting, habitual, pinhole, or BCVA. Where both eyes are available, the better-seeing eye will be used for patient-level analyses. Non-standard measures (counting fingers, hand movements, light perception, and NLP) will be retained as categorical measures.

#### 2.5.2 Near visual acuity and Reading performance

Near visual acuity and reading performance was assessed using MNREAD acuity chart, including reading distance, critical print size (M-units), reading time (seconds), and calculated required magnification, where available. The LEA near chart was used for paediatric patients and individuals unable to read standard MNREAD text. Aided measurements will be interpreted as immediate, in-clinic aided visual performance. Measurements obtained before and during or after an aid trial will be considered same-visit measurements.

#### 2.5.3 Objective and subjective refraction

Objective refraction will be extracted according to the method documented in the clinical record, including focimetry to determine existing prescribed aids, autorefraction, or retinoscopy, where performed. Subjective refraction results will also be extracted for distance and near where documented. Refraction results will be extracted as documented in the clinical record. For analysis, refractive status will be classified as myopia, hyperopia, astigmatism, or no significant refractive error based on the recorded subjective refraction results.

#### 2.5.4 Supplementary visual-function assessments

Additional visual function assessments will be extracted where documented, including contrast sensitivity, colour vision, visual field assessment, stereoacuity, cover test, and intraocular pressure. Contrast sensitivity was assessed using the Pelli-Robson chart, with results recorded as the log contrast sensitivity score. Color vision was assessed using Ishihara plates, with results recorded as normal/documented deficiency. Stereoacuity was assessed using the Randot Stereotest, with results recorded in arcseconds. Visual field assessment results will be extracted as documented in the clinical record. Where available, the recorded outcome will be classified as pass, fail, or other documented result for each eye. Cover test and intraocular-pressure findings will likewise be recorded according to the documented method and result.

#### 2.5.5 Low vision aid trials and immediate aided performance

Where a low vision aid was trialled during the index assessment, the type of aid, power or magnification, task for which it was trialled, and resulting visual performance will be extracted. Distance and near aided visual acuity will be retained separately from unaided measurements. The difference between unaided and same-visit aided visual acuity will be used to describe immediate aided visual performance among patients for whom both measurements are available.

Documentation of an aid trial, recommendation or prescription will be distinguished from documentation of actual aid dispensing. A recommendation or prescription will not be interpreted as evidence that the aid was subsequently dispensed or used outside the clinical setting.

### 2.6 Classification of diagnosis

Diagnoses will be recorded at the patient level, although diagnoses for the right and left eyes will be retained separately where documented. Where both eyes have the same diagnosis, this will be assigned as the principal diagnosis. Where bilateral diagnoses differ, the principal diagnosis will be based on the treating clinician's documented assessment of the main contributor to the patient's visual impairment.

Where multiple ocular conditions are documented and no single principal cause is explicitly identified, conditions will be prioritized using the following hierarchy: (1) the condition documented as the primary diagnosis by the treating clinician; (2) the condition judged to have the greatest contribution to the patient's visual impairment based on the documented clinical findings; and (3) where a principal cause cannot be established reliably, the patient will be classified as having multiple contributing ocular conditions.

Diagnostic categories will be coded using the ICD-10 where a corresponding diagnosis can be established from the clinical record. Conditions will additionally be classified by aetiological category including congenital/hereditary (present from birth or genetically determined), acquired (developing after birth due to disease, trauma, or degeneration), or indeterminate (insufficient documentation to classify), based on the diagnosis and any documented history.

Where applicable, causes will also be categorized as avoidable or unavoidable based on the prespecified study classification. Avoidable causes will include conditions for which visual loss is considered preventable or treatable through timely and appropriate intervention, whereas unavoidable causes will include conditions for which currently available interventions would not reasonably be expected to prevent the underlying visual loss. Where the available record does not contain sufficient information to make this distinction reliably, the cause will be classified as indeterminate.

### 2.7 Study endpoints

#### 2.7.1 Primary endpoint

The primary endpoint will be the proportion of eligible patients with a documented recommendation/prescription for at least one low vision aid at the index assessment (see Table 2).

**Table 2:** Study endpoints.

Endpoint	Operational definition	Time point	Unit	Denominator
Aid recommendation/ prescription	Documented recommendation/prescription of ≥1 low vision aid	Index visit	Yes/no or aid category	All eligible patients assessed
Aid dispensing	Documented dispensing of ≥1 recommended aid	After prescription	Yes/no	Patients with an aid recommended
Aided acuity	Acuity measured using selected device	In-clinic trial	logMAR	Patients completing trial
Diagnosis	Primary ocular diagnosis assigned	Index visit	Category	All eligible patients
Referral source	Referring facility or direct presentation	Same visit	Category	All eligible patients
Service volume	Number of eligible patient assessments	Monthly/annual	Number of patients	__

#### 2.7.2 Secondary endpoints

- **Aided visual acuity change:** The difference between unaided and aided visual acuity (distance and/or near, better-seeing eye), where both measurements are available. Measurements will be classified according to whether they were obtained during the initial assessment/aid trial or at a subsequent documented visit.
- **Diagnosis distribution:** the primary ocular diagnosis assigned at the index encounter.
- **Referral source:** the referring facility (or self/direct presentation) recorded at the index encounter.
- **Aid type prescribed:** the category of low vision aid(s) recommended (optical, non-optical, electronic).
- **Service volume over time:** the number of eligible patient assessments recorded by centre and calendar period during the study period.

### 2.8 Sample size

A census approach will be used, whereby all eligible records identified at the four participating centres during the study period will be included. Approximately 100 eligible patient records are anticipated, comprising approximately 40 records from the tertiary centre and 20 records from each of the three secondary centres. The final sample size will be determined by the number of eligible records available after application of the predefined inclusion and exclusion criteria.

Based on the anticipated distribution of records, approximately 20–40 patients may be expected to receive a documented recommendation or prescription for at least one low vision aid, assuming that approximately 20%–40% of eligible patients receive an aid recommendation. The numbers recommended in each aid category will depend on the observed distribution of aid prescribing in the records. Similarly, the number of patients with complete data for the primary endpoint will depend on the completeness of documentation of aid recommendation or prescription.

Regression analyses will be conducted only where the number of outcome events and completeness of relevant covariates are sufficient. The number of predictors included in any multivariable model will be limited according to the effective sample size and number of observed outcome events. Where the available data are insufficient to support reliable multivariable estimation, analyses will be restricted to descriptive and appropriately specified univariable analyses.

### 2.9 Statistical analysis

#### 2.9.1 Descriptive analysis

Data will be transferred to R (version 4.5.0) for analysis. Analyses will be conducted at the patient level using the index low vision assessment. Demographic and clinical characteristics will be summarized overall and by centre. Continuous variables will be presented as means and standard deviations or medians and interquartile ranges, as appropriate, while categorical variables will be summarized as frequencies and percentages.

Temporal trends in service volume will be summarized by centre and active facility-month to account for differences in the timing and duration of centre operation. Frequencies and proportions of low vision aid recommendation/prescription and dispensing will be reported overall and by centre. Optical, non-optical, and electronic aids will be treated as non-mutually exclusive categories because patients may receive more than one type of aid. Referral sources and diagnostic categories will also be summarized. Effect estimates will be reported with 95% confidence intervals (CIs), where appropriate.

#### 2.9.2 Inferential analysis

The primary outcome, recommendation/prescription of at least one low vision aid, will be analysed as a binary outcome (yes/no). Centre will be included as a fixed effect where sample size permits, with centre-stratified analyses also presented. Multivariable regression models will be considered only where the number of outcome events and completeness of relevant covariates are sufficient. Predictors will be selected *a priori* based on clinical relevance rather than automated stepwise selection, and the number of predictors will be limited according to the available sample size and outcome events.

Optical, non-optical, and electronic aid categories will be analysed separately where sufficient events are available. Immediate aided visual performance will be assessed among patients with both unaided and same-visit aided visual-acuity measurements. Change will be calculated as unaided minus aided logMAR, with positive values indicating improvement. Where appropriate, near visual acuity and reading-performance measures will be analysed similarly.

Multivariable models will assess non-linearity, multicollinearity, model fit, and influential observations using methods appropriate to the fitted model. Clinically justified interactions will be considered where supported by the data. Secondary analyses will be considered exploratory, with emphasis placed on effect estimates and 95% CIs.

#### 2.9.3 Missing data

Missingness will be reported for each variable, overall, by centre, and by calendar period. Where available, reasons will be classified as not recorded, not performed, not clinically indicated, unavailable at the centre, patient unable to complete the test, or not applicable. Structural missingness due to unavailable services will be distinguished from incomplete recording.

Missing data will be handled according to their extent, pattern, and likely mechanism rather than a fixed threshold. Complete-case analysis will be used where appropriate. Multiple imputation will be considered when clinically and statistically appropriate, using the outcome, relevant demographic and clinical variables, centre, and variables associated with missingness. The number of imputed datasets will reflect the extent of missing information, with convergence and plausibility assessed. Imputed results will be compared with complete-case and sensitivity analyses. Primary outcome data will not be imputed when missing documentation cannot distinguish absence of the outcome from missing recording.

#### 2.9.4 Bias and generalisability

Several sources of bias are possible in this facility-based retrospective record review. Referral and selection bias may arise because the study includes only patients who were referred to and attended the four participating centres. Individuals unaware of low vision services, unable to travel, screened but not referred, referred but not attending, or living outside the network catchment area will not be represented. Missing-record bias may arise from incomplete or unavailable records. Information bias may result from incomplete or inconsistent documentation of clinical assessments, aid recommendations, and dispensing. Differences in measurement procedures and documentation practices between centres and over time may introduce inter-centre measurement variation and temporal documentation bias. Diagnostic misclassification may occur where diagnoses are uncertain or incompletely documented.

These risks will be addressed through standardized data extraction, prespecified and consistent diagnostic classification rules, and transparent reporting of missing data. As the study has no longitudinal follow-up, unequal follow-up is not applicable.

The findings will characterize the demographic and clinical characteristics, referral patterns, low vision aid prescribing, and documented clinical and service indicators among patients who accessed the four participating centres. Generalisability will therefore be limited to patient populations accessing similar low vision rehabilitation services and should not be assumed for the broader Ghanaian population.

### 2.10 Ethics and dissemination

Ethical approval for this study has been obtained from the Committee on Human Research, Publication and Ethics (CHRPE), Kwame Nkrumah University of Science and Technology, under reference CHRPE/AP/752/26. Given the retrospective nature of this study and its reliance on routinely collected clinical records, the Committee also granted a waiver of individual patient consent for the use of these records. As of the date of this protocol, data extraction has not yet begun; no records have been extracted, no outcome distributions have been examined, and no statistical analysis has been undertaken. This protocol is being submitted and registered prior to the start of data extraction. We plan to publish our results in a peer-reviewed journal and present findings at academic conferences.

### 2.11 Data management and confidentiality

Data will be entered into a secure, password-protected Microsoft Excel database hosted at KNUST. Each patient will be assigned a unique study identification code, and direct identifiers such as names will be excluded from the analytical dataset. Where required, the linkage file will be stored separately in a password-protected location accessible only to authorized study personnel.

Access will be restricted to authorized members of the research team. Where supported by the institutional system, data will be encrypted at rest and during transfer. Data will not be transferred outside the participating institutions or Ghana unless approved by the relevant institutional and ethical authorities; any approved transfer will use secure, encrypted methods and exclude direct identifiers.

Data will be retained for the period required by KNUST institutional and ethical requirements and then securely deleted or destroyed in accordance with institutional procedures. Data cleaning, corrections, and changes will be documented to maintain an audit trail, with the original extracted data preserved separately from cleaned and analysis-ready datasets.

## 3 Discussion

This protocol describes a multicentre retrospective record review of patients accessing low vision rehabilitation services across four centres in Ghana. The study will characterize patient demographic and clinical profiles, including diagnoses, referral sources, low vision aid recommendation and dispensing, and immediate aided visual performance. The multicentre design will also allow description of differences in case mix, referral patterns, prescribing practices, and service volume between participating centres.

To our knowledge, this will be the first multicentre evaluation in Ghana to describe these aspects of low vision service delivery across multiple rehabilitation centres. For the HM KNUST Low Vision Project, the findings may identify differences in patient characteristics, referral patterns, aid provision, and service volume that can inform service planning and data-system development. For the Ghana Health Service and Ministry of Health, the study may provide evidence to support the development and strengthening of low vision rehabilitation services. For the scientific community, the study will add to a very sparse literature on low vision service delivery in West Africa, where systematic outcome data is almost entirely absent (22). More broadly, the study will address a gap that has direct implications for global eye health targets. The World Report on Vision (2019) (42), the WHO Global Action Plan for Eye Care (43), and the WHO Planning guide for integrated vision rehabilitation (36), identify low vision rehabilitation as a priority area for LMICs, and call specifically for better evidence on service delivery models and patient outcomes in these settings. To this end, this study is designed to address the evidence gap.

Several limitations should be considered. The retrospective design depends on the completeness and accuracy of existing records and may be affected by missing and structurally unavailable data, inter-centre differences in measurement and documentation, and diagnostic misclassification. The facility-based sample includes only patients who successfully accessed the participating centres and therefore may be affected by selection and referral bias. The absence of primary-centre denominators limits interpretation of referral patterns, while incomplete device-dispensing documentation may affect estimates of aid provision. Limited functional and patient-reported outcomes restrict assessment of broader rehabilitation outcomes. Although follow-up information may be available for some records, it is not systematically collected and will not support conclusions about long-term outcomes. Finally, the absence of a comparison group or counterfactual means that observed differences between centres or over time cannot be attributed causally to the HM KNUST programme.

## 4 Conclusion

This study will characterize the demographic and clinical profiles of patients accessing low vision services at four centres in Ghana, and evaluate documented referral sources, low vision aid provision, and prespecified clinical and service outcomes. The findings may identify documentation and service delivery gaps and inform the design of future prospective evaluations of the wider low vision referral network. By establishing a standardized approach to reviewing routinely collected low vision records, this protocol also offers a replicable framework that other low vision services in Ghana and similar settings may adapt for their own service evaluation.
