# Supplementary material for: Clinical profiles, referral patterns and low vision aid prescribing across four rehabilitation centres in Ghana: protocol for a multicentre retrospective record review
Source: Front Med (Lausanne). 2026 Oct 9;13:1936778. doi: 10.3389/fmed.2026.1936778 (PMC13690620; doi:10.3389/fmed.2026.1936778)
Supplement: Supplementary file 1 [file Table1.docx]

**HM KNUST LOW VISION PROJECT PATIENT FORM**

**PART 1 – INITIAL ASSESSMENT**

**Name of Facility:**

**Name of Referring Facility:**

**SECTION 1: PATIENT DEMOGRAPHIC DATA**

Name: ………………………………………………. Date…..…..……20……

Date of Birth:…………………………………	Sex: Male/Female

Phone No:…………………………….... Occupation…..…………………..

Residence: ……..…………………	Hobby(ies)………………………..

**SECTION 2: CASE HISTORY AND VISUAL ACUITY**

CC/C(HPC):

ODQ:

POH:

PMH:

FOH:

FMH:

SH:

Meds/Allergies:

**SECTION 3: GENERAL EXAMINATION**

BP: …………/…………mmHg

| VISUAL ACUITY | CHART USED |  | OD | OS | OU |
| --- | --- | --- | --- | --- | --- |
| **DISTANCE** |  | SC |  |  |  |
|  |  | CC/PH |  |  |  |
| **NEAR** |  | SC |  |  |  |
|  |  | CC |  |  |  |

Distance Used…………………..

Eccentric fixation?…………………

Interpupillary Distance (PD): ………. (far)/……….(near)mm

Objective Refraction

Keratometry

| K  Reading | OD | | OS | |
| --- | --- | --- | --- | --- |
| Diopters | ……@…… | ……@…… | ……@…… | ……@…… |
| mm | ……@……. | ……@...… | ……@……. | ……@….. |

Focimetry

| Eye | Sphere | Cyl | Axis |
| --- | --- | --- | --- |
| OD |  |  |  |
| OS |  |  |  |

Autorefraction

| Eye | Sphere | Cyl | Axis |
| --- | --- | --- | --- |
| OD |  |  |  |
| OS |  |  |  |

Retinoscopy

Power Cross:

Working distance: ………………D

Gross: OD:

OS:

Net: OD: VA:

OS: VA:

Wet/Dry (please circle)

Subjective Refraction

| Eye | Sphere | Cyl | Axis | VA | ADD | NVA |
| --- | --- | --- | --- | --- | --- | --- |
| OD |  |  |  |  |  |  |
| OS |  |  |  |  |  |  |

Notes:

**SECTION 4: EXAMINATION, DIAGNOSIS AND MANAGEMENT**

Slit Lamp Examination:

OD	OS

……………………………Lids/lashes.…………………………………

……………………………Sclera……………………………………

……………………………Conjunctiva.…………………………………

……………………………Cornea.……………………………………

……………………………AC/Angle.…………………………………

………………………………Iris.………………………………………

……………………………Pupil.……………………………………

………………………………Lens.………………………………………

Internal Examination:

……………………………Vitreous………….………………………

…………………………….…Disc size.……………………………………

…………………………	Disc margin…………………………………

…………………	Disc colour.…………………………

……………………………Cupping(H/V).…………………………………

…………………ISNT Rule…………………………

…………………………Peripapillary region…………………………………

……………………………Nerve fibre layer…………………………………

………………………..……A/V ratio……………………………………

……………………………Macula/fovea……………………………………

…………………..….…Peripheral retina………………………………

Other Notes:

IOP Assessment:

OD: …………..mmHg	OS: ………….mmHg	Time: ……………

Tonometer used: ……………………

Impression:

Plan:

**SECTION 2: NEEDS ASSESSMENT**

Current Assistive Devices:

Daily Living Activities:

Goals:

**SECTION 3: VISUAL ACUITY AND REFRACTION**

| VISUAL ACUITY | CHART USED |  | OD | OS | OU |
| --- | --- | --- | --- | --- | --- |
| **DISTANCE** |  | CC |  |  |  |
|  |  |  |  |  |  |
| **NEAR** |  | CC |  |  |  |
|  |  |  |  |  |  |

Eccentric fixation?…………………

Distance Used………………..

READING PERFORMANCE

| CRITICAL PRINT SIZE (CPS) IN M-UNITS |  |
| --- | --- |
| READING SPEED IN WORDS PER MINUTE (wpm) |  |
| REQUIRED MAGNIFICATION |  |

Distance Used …………….

OBJECTIVE REFRACTION

Keratometry

| K  Reading | OD | | OS | |
| --- | --- | --- | --- | --- |
| Diopters | ……@…… | ……@…… | ……@…… | ……@…… |
| mm | ……@…… | ……@…… | ……@ .… | ……@ ...… |

Focimetry

| Eye | Sphere | Cyl | Axis |
| --- | --- | --- | --- |
| OD |  |  |  |
| OS |  |  |  |

Autorefraction

| Eye | Sphere | Cyl | Axis |
| --- | --- | --- | --- |
| OD |  |  |  |
| OS |  |  |  |

Retinoscopy

Power Cross:

Working distance: ………………D

Gross: OD:

OS:

Net: OD: VA:

OS: VA:

Wet/Dry (please circle)

SUBJECTIVE REFRACTION

| Eye | Sphere | Cyl | Prism | Axis | VA | ADD | NVA |
| --- | --- | --- | --- | --- | --- | --- | --- |
| OD |  |  |  |  |  |  |  |
| OS |  |  |  |  |  |  |  |

Notes:

**SECTION 4: SUPPLEMENTARY TESTS**

CONTRAST SENSITIVITY ASSESSMENT

| Contrast | OD | OS | OU |
| --- | --- | --- | --- |
| CC |  |  |  |

Test used ……………..

COLOUR VISION ASSESSMENT

Results:

Diagnosis: Protan

Deutan

Tritan

Indeterminate

Severity (circle): mild moderate severe	Test used: ………………….

VISUAL FIELD ASSESSMENT (PLEASE INDICATE TEST USED)

Confrontation Visual Fields

Amsler Grid Assessment

Automated Perimetry

BINOCULAR VISION ASSESSMENT

Extraocular Motility (Broad H) Test (*Please draw from the patient’s view)*

Patient responses

Pains	direction(s) …………………..

Discomfort	direction (s)…………………..

Diplopia (direction (s))	worst at ……………….

Impressions from EOMs:

Stereoacuity

Local: ………..secs Global: …………..secs	Test used: …………………

Cover Test

|  | Lateral (∆) | Vertical (∆) | Torsional (∆) |
| --- | --- | --- | --- |
| Distance |  |  |  |
| Near +1.00Ds |  |  |  |
| Near -1.00Ds |  |  |  |
| Near +2.50/+3.00Ds |  | Gradient AC/A: |  |

Impression/Comments:

**SECTION 5: AIDS SELECTION AND RECOMMENDATION**

| Type of Aid | Power/Magnification | Task | Performance |
| --- | --- | --- | --- |
|  |  |  |  |
|  |  |  |  |

Indicate any non-optical aid recommended:

Task recommended for:

Results:

Other Recommendations:

Overall impression/Diagnosis:

Treatment/Management plan (including reviews and referrals)

Attending Clinician name and sign……………………..….

Date……………………

**OTHER TESTS AND COMMENTS**
