# Supplementary material for: Clinical profiles, referral patterns and low vision aid prescribing across four rehabilitation centres in Ghana: protocol for a multicentre retrospective record review
Source: Front Med (Lausanne). 2026 Oct 9;13:1936778. doi: 10.3389/fmed.2026.1936778 (PMC13690620; doi:10.3389/fmed.2026.1936778)
Supplement: Supplementary file 2 [file Table2.docx]

**Supplementary File 2: Variable Dictionary and Coding Manual**

**General Coding Principles**

- Data will be extracted only from information documented in the eligible clinical record.
- Information will not be inferred when it is not explicitly documented.
- Each patient will be assigned a unique study identification number.
- Names and other direct identifiers will not be entered into the analytical dataset.
- Categorical variables will be coded using predefined numerical or text codes.
- Missing information will be distinguished from information documented as "not applicable" or "unknown."
- Original clinical measurements will be retained where available, alongside standardized/derived values.

**Missing-Data Codes**

| **Code** | **Meaning** | **Use** |
| --- | --- | --- |
| 99 | Not documented/missing | Variable expected but information absent |
| 98 | Unknown | Record explicitly states that information is unknown |
| 97 | Not applicable | Variable does not apply to the patient |
| 96 | Unable to determine | Information is present but cannot be reliably classified |
| 0 | No/absent | Used only for variables where zero represents a valid response |

These codes will not be treated as numerical measurements during statistical analysis.

**Patient and Administrative Variables**

| **Variable** | **Definition** | **Type** | **Coding** |
| --- | --- | --- | --- |
| Study_ID | Unique identifier assigned to each eligible record | Categorical | Sequential ID |
| Centre | Rehabilitation centre where low vision assessment occurred | Categorical | 1=Centre 1; 2=Centre 2; 3=Centre 3; 4=Centre 4 |
| Assessment_date | Date of index low-vision assessment | Date | DD/MM/YYYY |
| Age | Age in completed years at index assessment | Continuous | Years |
| Sex | Sex documented in the clinical record | Categorical | 1=Male; 2=Female; 99=Missing |
| Residence | Patient's documented place of residence | Categorical/text | Recorded as documented |
| Occupation | Main occupation documented at assessment | Categorical/text | Recorded as documented |

**Referral Variables**

| **Variable** | **Definition** | **Coding** |
| --- | --- | --- |
| Referral_status | Whether the patient was referred to the low vision service | 0=No; 1=Yes |
| Referral_source | Facility/person/service from which patient was referred | 1=Eye clinic; 2=Hospital/medical facility; 3=Optometrist; 4=Ophthalmologist; 5=Community/NGO; 6=Self-referral; 7=Other; 99=Missing |

**Clinical Variables**

| **Variable** | **Definition** | **Coding** |
| --- | --- | --- |
| Chief_complaint | Main visual complaint documented at assessment | Text/categorized |
| Ocular_history | Relevant previous ocular history | Text |
| Systemic_history | Relevant systemic medical history | Text |
| Family_history | Relevant family history | 0=None documented; 1=Present; 98=Unknown; 99=Missing |
| Primary_diagnosis | Main ocular/visual diagnosis documented | Classified according to Supplementary File 3 |
| Secondary_diagnosis | Additional diagnosis documented | Classified according to Supplementary File 3 |

**Visual-Acuity Variables**

| **Variable** | **Definition** | **Unit** |
| --- | --- | --- |
| Distance_VA_OD_unaided | Unaided distance VA of right eye | logMAR |
| Distance_VA_OS_unaided | Unaided distance VA of left eye | logMAR |
| Distance_VA_OD_best | Best-corrected distance VA of right eye | logMAR |
| Distance_VA_OS_best | Best-corrected distance VA of left eye | logMAR |
| Near_VA_unaided | Unaided near VA | Original notation |
| Near_VA_best | Best-corrected near VA | Original notation |
| Distance_VA_aided | Distance VA using prescribed/recommended aid | logMAR |
| Near_VA_aided | Near VA using prescribed/recommended aid | logMAR |

**Refraction Variables**

| **Variable** | **Definition** |
| --- | --- |
| Objective_refraction | Objective refractive findings documented in the record |
| Subjective_refraction | Final subjective refraction documented |
| Sphere_OD/OS | Spherical refractive error |
| Cylinder_OD/OS | Cylindrical refractive error |
| Axis_OD/OS | Cylinder axis |
| Add_OD/OS | Near addition where documented |

**Low Vision Aid Variables**

| **Variable** | **Definition** | **Coding** |
| --- | --- | --- |
| Aid_recommended | Whether ≥1 low-vision aid was documented as recommended | 0=No; 1=Yes; 99=Missing |
| Aid_prescribed | Whether ≥1 aid was documented as prescribed | 0=No; 1=Yes; 99=Missing |
| Aid_dispensed | Whether the record documents that the aid was actually dispensed/provided | 0=No; 1=Yes; 98=Unknown; 99=Missing |
| Aid_type | Type of aid recommended/prescribed | Classified according to Supplementary File 3 |
| Number_of_aids | Number of aids recommended/prescribed | Integer |
| Multiple_aid_categories | Whether aids from >1 category were prescribed | 0=No; 1=Yes |

**Important distinction:** A documented recommendation or prescription will not be interpreted as evidence that an aid was dispensed unless dispensing is explicitly documented.

**Derived Variables**

| **Variable** | **Definition** |
| --- | --- |
| Better_eye_unaided_logMAR | LogMAR VA of the better-seeing eye at the index assessment |
| Better_eye_aided_logMAR | LogMAR VA of the better-seeing eye with the aid |
| Aided_VA_change | Difference between unaided and same-visit aided VA |
| Aid_prescription_any | Binary indicator for ≥1 documented low-vision aid prescription/recommendation |
| Diagnosis_group | Standardized diagnosis category according to Supplementary File 3 |
| Referral_group | Standardized referral-source category |

For visual acuity, a positive improvement score will be defined as:

**Unaided logMAR − aided logMAR**

Thus, a positive value indicates improved visual acuity with the aid.

**Quality-Control Rules**

Before analysis:

1. Range checks will be performed for continuous variables.
2. Impossible or implausible values will be compared with the source record.
3. A random sample of records will undergo independent verification by a second trained reviewer.
4. Discrepancies will be resolved by reference to the original clinical record and, where necessary, discussion between reviewers.
