# Supplementary material for: Clinical profiles, referral patterns and low vision aid prescribing across four rehabilitation centres in Ghana: protocol for a multicentre retrospective record review
Source: Front Med (Lausanne). 2026 Oct 9;13:1936778. doi: 10.3389/fmed.2026.1936778 (PMC13690620; doi:10.3389/fmed.2026.1936778)
Supplement: Supplementary file 3 [file Table3.docx]

**Supplementary File 3: Diagnostic Classification, Visual-Acuity Conversion and Low-Vision Aid Classification Rules**

**Part A. Diagnostic Classification Rules**

**1. Diagnostic Categories**

| **Category** | **Examples of diagnoses included** |
| --- | --- |
| Refractive error | Myopia, hyperopia, astigmatism, presbyopia |
| Cataract | Age-related, congenital, traumatic or other documented cataract |
| Glaucoma | Primary open-angle, angle-closure, congenital or secondary glaucoma |
| Retinal disease | Diabetic retinopathy, age-related macular degeneration, retinal dystrophy, retinal detachment and other retinal disorders |
| Optic nerve disorders | Optic atrophy, optic neuropathy and other documented optic nerve disorders |
| Corneal/anterior segment disorders | Corneal opacity, keratopathy and other anterior segment disorders |
| Ocular trauma | Visual impairment attributed primarily to ocular trauma |
| Congenital/developmental conditions | Congenital ocular abnormalities and developmental visual disorders |
| Neurological/cerebral visual impairment | Cortical/cerebral visual impairment and other documented neurological causes |
| Multiple conditions | Two or more clinically relevant conditions without a clearly documented primary cause |
| Other | Diagnosis not fitting the predefined categories |
| Unspecified | Visual impairment documented but no specific diagnosis recorded |

**2. Primary Diagnosis**

When multiple diagnoses are documented, the diagnosis explicitly identified by the clinician as the primary/main diagnosis will be coded as the primary diagnosis.

If no primary diagnosis is specified, the condition judged from the record to be the principal cause of the patient's visual impairment will be coded only when this can be determined reliably from the documentation. Otherwise, the case will be coded as **multiple conditions** or **unspecified**, as appropriate.

No diagnosis will be inferred solely from visual-acuity findings.

**Part B. Visual-Acuity Rules**

**1. ETDRS Measurements**

Where visual acuity is recorded directly in logMAR or ETDRS letter scores, the original measurement will be retained.

**2. Counting Fingers, Hand Movements and Light Perception**

Non-numeric visual-acuity measurements will be retained in their original form.

Where a standardized logMAR value is required for analysis, a prespecified conventional conversion will be applied consistently across all records. Such converted values will be flagged as derived rather than directly measured.

If a reliable conversion cannot be made, the original category will be retained and the value will not be included in analyses requiring continuous logMAR measurements.

**3. No Light Perception**

No light perception (NLP) will not be treated as an ordinary numeric Snellen measurement.

NLP will be retained as a categorical value and will be excluded from analyses requiring a continuous logMAR value unless a prespecified analytical method is justified.

**4. Better-Seeing Eye**

For analyses involving the better-seeing eye, the eye with the lower (better) logMAR value at the specified assessment will be classified as the better-seeing eye.

If both eyes have equal visual acuity, either eye may be selected according to the prespecified database rule, provided that the same rule is applied to all records.

**Part C. Low-Vision Aid Classification Rules**

**1. Aid Categories**

| **Category** | **Examples** |
| --- | --- |
| Optical distance aids | Telescopes and distance magnifiers |
| Optical near aids | Hand magnifiers, stand magnifiers, spectacle-mounted magnifiers, high-add spectacles |
| Electronic/digital aids | Electronic magnifiers, digital magnification devices and screen-based magnification devices |
| Non-optical aids | Large-print materials, writing guides, typoscopes, task lighting and contrast-enhancing devices |
| Environmental/adaptive aids | Environmental modifications and adaptations intended to improve visual functioning |
| Other | Aid that cannot be assigned to the predefined categories |

**2. Multiple Aids**

If more than one aid is documented, each aid will be recorded separately where the database permits. A patient-level variable indicating whether the patient received/recommended aids from multiple categories will also be generated.

**3. Unclear Aid Information**

If an aid is mentioned but its type cannot be determined reliably, it will be coded as **Other/Unclassified** rather than inferred from context.
