# Supplementary material for: Clinical profiles, referral patterns and low vision aid prescribing across four rehabilitation centres in Ghana: protocol for a multicentre retrospective record review
Source: Front Med (Lausanne). 2026 Oct 9;13:1936778. doi: 10.3389/fmed.2026.1936778 (PMC13690620; doi:10.3389/fmed.2026.1936778)
Supplement: Supplementary file 4 [file Table4.docx]

**Supplementary File 4: Prespecified Statistical Analysis Plan**

**1. Unit of Analysis**

The primary unit of analysis will be the individual patient.

Each eligible patient will contribute one index low-vision assessment to the primary analysis. Where multiple visits are documented, the earliest eligible low-vision assessment during the study period will be considered the index assessment.

Subsequent visits will be used only for analyses of documented follow-up.

**2. Descriptive Analysis**

Categorical variables will be summarized using frequencies and percentages.

Continuous variables will be assessed for distribution using graphical and numerical methods.

Approximately normally distributed continuous variables will be summarized using mean and standard deviation.

Skewed continuous variables will be summarized using median and interquartile range.

Age and other relevant continuous variables may also be categorized where clinically meaningful.

**3. Visual Acuity Analysis**

For paired same-visit observations, the change in visual acuity will be calculated as:

**Change = unaided logMAR − aided logMAR**

Positive values indicate improvement with the aid.

Paired comparisons will be performed only among patients with both valid unaided and aided measurements.

Because lower logMAR values represent better visual acuity, interpretation of effect direction will account for this scale.

**4. Analysis of Low Vision Aid Provision**

The frequency and proportion of patients with:

- a documented recommendation/prescription;
- documented dispensing; and
- no documented aid provision

will be reported.

The type of aid will be summarized using the standardized categories in Supplementary File 3.

Recommendation/prescription and dispensing will not be considered equivalent outcomes.

**5. Factors Associated With Aid Recommendation/Prescription**

Exploratory analyses will examine whether selected patient and clinical characteristics are associated with the primary outcome.

Potential predictors will be selected a priori based on clinical relevance and the study objectives rather than by automated backward stepwise selection.

Candidate variables may include:

- age;
- sex;
- diagnosis category;
- visual-acuity category;
- referral source;
- centre;
- and other clinically justified variables with adequate data.

Given the expected sample size of approximately 100 records, the number of predictors included in multivariable models will be limited to reduce overfitting.

Effect estimates will be reported with 95% confidence intervals.

**6. Centre-Level Analysis**

Differences across the four participating centres will be described using centre-specific frequencies and proportions and, where appropriate, comparative statistical tests.

Centre will be considered as a fixed effect in regression models where adjustment for centre is required.

Cluster-robust standard errors will not be relied upon as the primary approach because only four centres are included.

Where sample sizes permit, centre-stratified analyses will be presented.

The study will not make population-level claims based on differences between centres.

**7. Missing Data**

The extent of missingness will be reported for all key variables.

Missing information will be classified, where possible, as:

- not documented;
- unknown;
- not applicable; or
- unable to determine.

The proportion of missing observations will be reported for the primary outcome and major covariates.

No missing value will be replaced simply because it is absent from the record.

Variables with substantial missingness will not automatically be imputed. The potential impact of missing information on interpretation will be discussed.

Complete-case analysis will be used for analyses requiring variables that are unavailable for some records, unless an alternative approach is prespecified and justified.

**8. Statistical Significance**

For inferential analyses, statistical significance will be assessed using a two-sided alpha level of 0.05.

Effect estimates will be presented with 95% confidence intervals.

Because the study is primarily descriptive and exploratory, emphasis will be placed on effect estimates, confidence intervals, and clinical interpretation rather than statistical significance alone.

**9. Software**

Data cleaning and statistical analyses will be conducted using R statistical software (version to be specified at the time of analysis).

All data transformations and analytical decisions will be documented to facilitate reproducibility.

**10. Reporting**

Results will be reported in accordance with the objectives and prespecified outcomes.

The findings will be interpreted as describing the population of patients accessing services at the four participating rehabilitation centres.
